# Evaluation of young smokers and non-smokers with Electrogustometry and Contact Endoscopy

**DOI:** 10.1186/1472-6815-9-9

**Published:** 2009-08-20

**Authors:** Pavlidis Pavlos, Nikolaidis Vasilios, Anogeianaki Antonia, Koutsonikolas Dimitrios, Kekes Georgios, Anogianakis Georgios

**Affiliations:** 1 2nd ENT Department of Aristotle University of Thessaloniki, Thessaloniki, Greece; 2 Laboratory of Experimental Physiology, Aristotle University of Thessaloniki, Thessaloniki, Greece

## Abstract

**Background:**

Smoking is the cause of inducing changes in taste functionality under conditions of chronic exposure. The objective of this study was to evaluate taste sensitivity in young smokers and non-smokers and identify any differences in the shape, density and vascularisation of the fungiform papillae (fPap) of their tongue.

**Methods:**

Sixty-two male subjects who served in the Greek military forces were randomly chosen for this study. Thirty-four were non-smokers and 28 smokers. Smokers were chosen on the basis of their habit to hold the cigarette at the centre of their lips. Taste thresholds were measured with Electrogustometry (EGM). The morphology and density of the fungiform papillae (fPap) at the tip of the tongue were examined with Contact Endoscopy (CE).

**Results:**

There was found statistically important difference (*p *< 0.05) between the taste thresholds of the two groups although not all smokers presented with elevated taste thresholds: Six of them (21%) had taste thresholds similar to those of non-smokers. Differences concerning the shape and the vessels of the fungiform papillae between the groups were also detected. Fewer and flatter fPap were found in 22 smokers (79%).

**Conclusion:**

The majority of smokers shown elevated taste thresholds in comparison to non-smokers. Smoking is an important factor which can lead to decreased taste sensitivity. The combination of methods, such as EGM and CE, can provide useful information about the vascularisation of taste buds and their functional ability.

## Background

Complete loss of taste is rather uncommon because the presence of four major afferent routes for taste provides substantial redundancy to the sensory communication for taste and a substantial back-up system in case of failure of any single nerve.

There are two categories of taste measurement, whole mouth and regional tests. A preliminary evaluation of a patient suffering from taste disorders can be performed with the use of colourless solutions of sweet, bitter, sour and salt. More sophisticated is regional chemogustometry whereby chemicals are applied to part of the tongue using a piece of filter paper or a cotton swab [1]. Regional chemogustometry can also be performed using closed chambers cemented to the tongue [2].

The simplest regional test for evaluation of taste is EGM. EGM was introduced in the clinical assessment of taste sensitivity during the 1950s [3,4]. Compared to tests based on chemical solutions, EGM is an efficient clinical tool, used in the evaluation of taste disorders caused by different factors such as middle-ear surgery [5], Bell's palsy [6], tumors, [7] and tonsillectomy [8]. Increased application of this method is due to its easiness, the short time required and its quantitative character.

CE is a diagnostic technique suitable for head and neck screening. It was developed for observing cell construction in the epithelial surface. The first application of CE was in gynecology [9]. The quality of the images and magnifications obtained with endoscopes, led to the application of CE in otolaryngology [10,11]. CE allows for both in vivo and in situ observations of pathology in the superficial layer of the tongue, nasal mucosa, vocal cords in laryngomicrosurgery and nasopharynx [12-15].

The effects of smoking on taste sensitivity [16] and olfaction [17] have been studied since the early 60's. However, up to day, only few experimental studies provide histological data about the effects of smoke on the size and shape of the tongue papillae. The aim of this study is to investigate if smokers and non-smokers differ in EGM thresholds on the anterior and posterior tongue and soft palate and if any observed difference, in EGM thresholds on the anterior tongue of smokers vs. non-smokers, can be attributed to a difference in the density or morphology of fungiform papillae at that site.

## Methods

Data from sixty-two randomly chosen healthy young males, aged 18–31 years (mean 24.87 y, SD ± 3.74 y), were taken into account in the study. Thirty-four of them were smokers and 28 non-smokers. None of them had received medication for any disease during the previous six months. No subject suffered from otolaryngological diseases, acute or chronic, such as rhinitis and sinusitis. Oral hygiene was also examined before testing. Concerning smokers, the duration of smoking was between 1 and 6 (3.2 ± 0.7) years and the number of cigarettes smoked ranged between 12 and 40 (18 ± 2.3) per day. All the smokers held their cigarettes, when smoking, at the centre of their lips. All the subjects were soldiers serving at the same military unit during the last three months and had similar nutritional habits. The majority of the examined persons were right-handed and only few of them (3 non-smokers and 1 smoker) were left-handed. They participated in the study only after they had been informed of its background and purpose and after their written consent was obtained. The protocol was reviewed and approved by the Institutional Review Board of The Aristotle University of Thessaloniki. Measurements were conducted according to the Helsinki Declaration.

In order to minimize variations in technique and interpretation of results, all examinations were carried out by the same researcher (PP). Before being tested, all the subjects were asked whether if they were experiencing any abnormal taste at the time. The checklist for recording their history is shown in Table 1.

**Table 1:** Checklist for history taking in patients complaining of taste problem

Was the taste problem sudden or gradual; bilateral or unilateral?
Can you taste anything or not?
Do you sense bad tastes without eating or when you eat something?
Have you ever noticed dry mouth or dry eye?
Have you ever noticed problems with your smell as well?

The list of questions to which all subjects answered before evaluation of taste thresholds and imaging. All non-smokers (n = 34) did not report any problem concerning their taste sensitivity. Three of the smokers (n = 28) reported bad tastes without eating something and 2 reported diminished sense of smell.

Taste sensitivity was evaluated with EGM. Electrical stimuli were delivered with an electrogustometer (TR-06, Rion Co, Japan) with a single, flat, circular stainless steel stimulus probe (5 mm diameter). The apparatus produces stimuli of low current and known durations (0.5, 1, 1.5 and 2 seconds). A feedback circuit controls the output current with an error of < 1% [18]. The thresholds varied with increases in the duration, though in many subjects the measurements concerning 1 and 1.5 seconds were the same.

All subjects had been instructed not to drink anything for an hour before the start of testing. Before measuring thresholds, a stimulus of 30 dB was administered to ensure that every subject could recognize electrogustometric stimuli. Stimulation started at the lowest stimulus strength (-6 dB) and increasingly stronger stimuli were presented until the subject recognized the stimulus. If the threshold for stimulus perception was not clearly distinguished, the next higher- and lower-strength stimuli were presented.

The electric threshold scores were measured at points on the right and left sides of the tongue apex (2 cm from the apex), the vallate papillae and the soft palate. Among healthy population electric thresholds for the apex, vallate papillae and soft palate are considered to be up to 8, 14 and 22 dB respectively [18]. Stimuli of all the available durations (0.5, 1, 1.5 and 2 seconds) were applied in order to investigate whether the different duration affected the recorded thresholds. Electric stimuli were applied, beginning at -8 dB to 34 dB (3–400 μÁ) in 2 dB steps. The relationship between the logarithmic control settings and the output currents is shown on Tables 2 and 3.

**Table 2:** Output Current dB Readings and Output Current

Output current dB	-6	-4	-2	0	2	4	6	8	10	12
Output current (μA)	4	5	6.4	8	10	13	16	20	25	32

The relationship between the logarithmic control settings and the output current. The output current can be adjusted in 2-dB steps from -6 dB to 12 Db.

**Table 3:** Output Current dB Readings and Output Current

Output current dB	14	18	20	22	24	26	28	30	32	34
Output current (μA)	50	64	80	100	130	160	200	250	320	400

The relationship between the logarithmic control settings and the output current. The output current can be adjusted in 2-dB steps from 14 dB to 34 Db.

We started measuring taste thresholds from right to left side. The threshold of the right side of the soft palate is ***Threshold A***, the one of the vallate papillae is ***Threshold B ***and the right side of the tongue apex ***Threshold C***. The corresponding thresholds of the left side of the tongue are ***Threshold D***, ***Threshold E ***and ***Threshold F ***(F for the left side of the soft palate). Cases where the threshold could not be measured were assigned as threshold value of 36 dB [19]. All 6 sites were tested with the same stimulus duration before proceeding to the next duration. In that way an interval of 3–4 minutes took place before the stimulus of the next duration was applied on a site, leading to a lower possibility of adaptation. The subjects had been instructed to recognize whether they perceived a sour/metallic taste suggesting gustatory function (taste threshold) or an electrical sensation suggesting trigeminal function.

Imaging was performed using a 30° contact endoscope (CE; magnification, × 60 and × 150; Karl Storz, Tuttlingen, Germany). Identification of fPap was performed at first by a noncontact technique. Subjects had rinsed their mouth with water prior to imaging. A contact technique was used without staining for imaging of subepithelial vessels. Methylene-blue 1% was used afterwards to stain epithelia and taste pores. Application of methylene blue was preceded by careful suctioning of the mucus. A filter paper strip delineating an area of 1 cm^2 ^was placed in a paramedian position on the tongue tip [20].

The subjects opened their mouth and the CE was placed on the methylene-blue stained surface of the tongue. To resolve the instability of the tongue during imaging the subjects were advised to hold the tip of the tongue with the upper and lower teeth, not too strongly in order to avoid stasis and hypaeremia which could lead to wrong estimation. They were also seated in a chair with their head and neck supported by a neck pillow. The form of the papillae and blood vessels were classified according to a previous classification [21]. The forms of the papillae were classified in **Type 1**, (egg-shaped or long ellipse type -No surface thickness), **Type 2 **(slight thick surface as compared to type 1), **Type 3 **(thick and irregular surface) and **Type 4 **(remarkably flat and atrophic surface). The classification of blood vessels was **Type A **(clear loop and wooden branch shape), **Type B **(unclear loop and wooden branch shape), **Type C **(elongated blood vessels), **Type D **(granular shape or dotted shape) and **Type E **(unclear blood vessels).

For estimating the density of fPap the best image from every individual was used. fPap could be distinguished from filiform papillae (which were stained dark), by their very light staining [22].

The results were analysed with SPSS 12 for Windows (SPSS Inc. Chicago, IL, USA).

The null hypothesis was that there was not a statistical difference between the subjects of the two groups. Non-parametric tests were applied because there was no normal distribution of the assessed thresholds. The level of statistical significance was set to *p *< 0.05. The thresholds of the two groups were compared using Kruskal-Wallis and Mann-Whitney tests. The Bonferroni correction was used where it was necessary. The same tests were used for the comparison between the thresholds recorded in each stimulus-duration in both groups.

## Results

All participants completed the study. Analysis of the answers of the checklist given before examination showed that 3 of the smokers reported bad tastes without eating or drinking something (phantogeusia) and 2 reported diminished sense of smell.

There were significant differences among the values of the six loci for smokers and non-smokers concerning all the durations of stimuli (0.5, 1, 1.5 and 2 sec). The results are shown in Tables 4 and 5.

**Table 4:** The mean, the standard deviation, the minimum and the maximum of taste thresholds, as recorded in all 6 loci for smokers(n = 28) and non-smokers(n = 34).

	**Smokers**				**Non-Smokers**			
	
	**Mean**	**Std. Deviation**	**Minimum**	**Maximum**	**Mean**	**Std. Deviation**	**Minimum**	**Maximum**
	
**thresholdA**	20.38	± 7.97	10	34	5.58	± 8.70	-2	14
**thresholdB**	21.13	± 7.00	12	34	3.23	± 9.77	-4	12
**thresholdC**	19.44	± 8.69	6	32	3.41	± 11.27	-4	12
**thresholdD**	23.25	± 7.68	12	36	7.00	± 12.04	0	16
**thresholdE**	23.63	± 6.39	10	36	9.41	± 9.55	2	18
**thresholdF**	21.88	± 7.93	10	36	6.43	± 8.95	0	16

It is obvious that smokers showed higher taste thresholds, even though the majority of them reported no taste disorder.

**Table 5:** Statistical differences between the taste thresholds of smokers (n = 28) and non-smokers (n = 34), as they were estimated with non-parametrical tests (Kruskal-Walis and Mann-Whitney U tests, *p *< 0.05).

	**Threshold A**	**Threshold B**	**Threshold C**	**Threshold D**	**Threshold E**	**Threshold F**
**0,5 sec (*p*)**	0,003	0,002	0,001	0,001	0,003	0,003
**1 sec (*p*)**	0,002	0,003	0,001	0,001	0,002	0,001
**1,5 sec (*p*)**	<0,001	<0,001	<0,001	<0,001	<0,001	0,003
**2 (*p*)**	0,002	0.001	<0,001	<0,001	0,001	0,003

It is interesting that only three of the smokers reported bad tastes without eating something, two reported diminished taste sense (hypogeusia) and two reported distortion of their taste sensitivity (dysgeusia). Significant differences were detected between the thresholds of the tongue's tip on the right and left sides of smokers' tongues, *p *= 0.002. The statistical difference between the thresholds of the right and left circumvallate papillae was also significant, *p *= 0.012. The difference between the thresholds of the right and left sides of the soft palate in smokers was insignificant. The thresholds on non-smokers' tongue and soft palate, as they have been recorded on the right and left side, differed significantly (Threshold A and F: *p *= 0.0021, Thresholds B and E: *p *= 0.045, Thresholds C and D: *p *= 0.038). It should be mentioned that taste thresholds varied in accordance with increases in duration. The median electrical taste threshold was higher in a 1-sec duration stimulus than in a 0.5-sec duration. In addition to the above finding, the thresholds did not tend to differ significantly in a 1-sec and in a 1.5-sec stimulus duration. The thresholds seemed to increase when we applied a 2-sec stimulus. The same findings were observed both in smokers and non-smokers and are presented in Tables 6 and 7.

**Table 6:** Statistical differences between 0.5 sec and 1 sec-duration stimulus (*p1*), 1 sec- and 1.5 duration stimulus (*p2*) and 1.5 sec- and 2 sec duration stimulus (*p3*) in smokers, as they were estimated with non-parametrical tests (Kruskal-Walis and Mann-Whitney U tests, *p *< 0.05).

	***A***	***B***	***C***	***D***	***E***	***F***
***P1***	**0,033**	**0,024**	**0,035**	**0,023**	**0,037**	**0,0043**
***P2***	0,046	0,048	0,048	0,046	0,041	0,041
***P3***	0,036	0,037	0,039	0,042	0,043	0,035

**Table 7:** Statistical differences between 0.5 sec and 1 sec-duration stimulus (*p1*), 1 sec- and 1.5 duration stimulus (*p2*) and 1.5 sec- and 2 sec duration stimulus (*p3*) in non-smokers, as they were estimated with non-parametrical tests (Kruskal-Walis and Mann-Whitney U tests, *p *< 0.05).

	***A***	***B***	***C***	***D***	***E***	***F***
***P1***	**0,035**	**0,031**	**0,033**	**0,037**	**0,045**	**0,0043**
***P2***	0,043	p > 0.05	0,046	0,048	0,045	0,044
***P3***	0,043	0,038	0,041	0,042	0,045	0,039

Six smokers (19%) showed normal taste thresholds in all of the loci tested. These subjects were examined additionally three times (2, 4 and 6 days after the first examination) and the results were the same as the first time.

**Figure 1:**
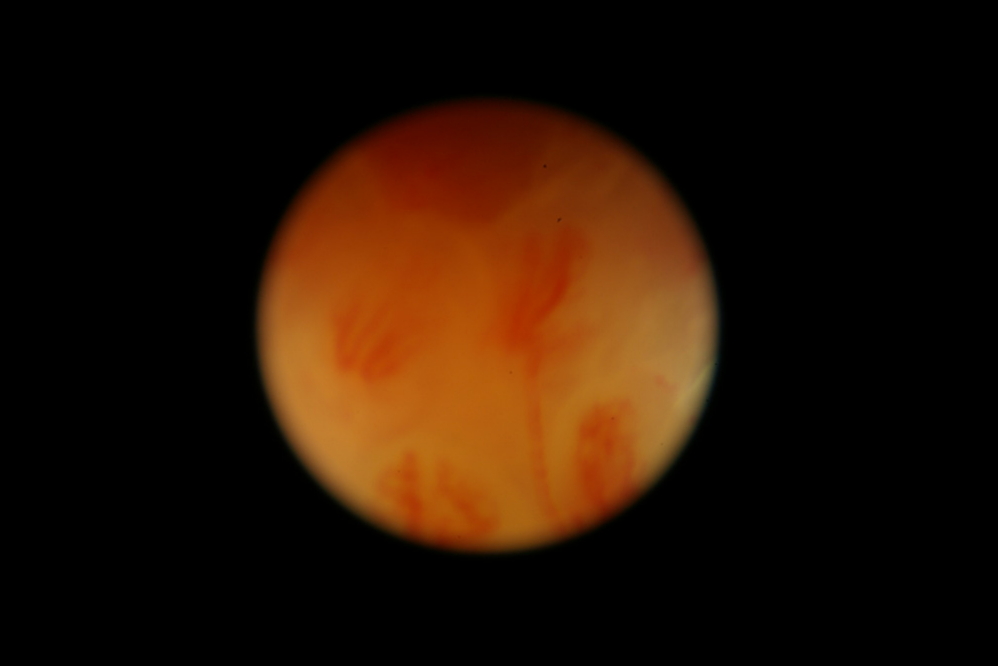
**Taste papillae of a non-smoker**. The blood vessels have wooden branch shape.

Differences in the shape and the vessels of the fPap between the two groups were also observed with CE. Using the classifications of a form papillae and blood vessels suggested in previous studies [21] non-smokers' papillae were found to belong to **Type 1 **(shape) and **Type A **(vascularisation) as seen on Figure 1.

**Figure 2:**
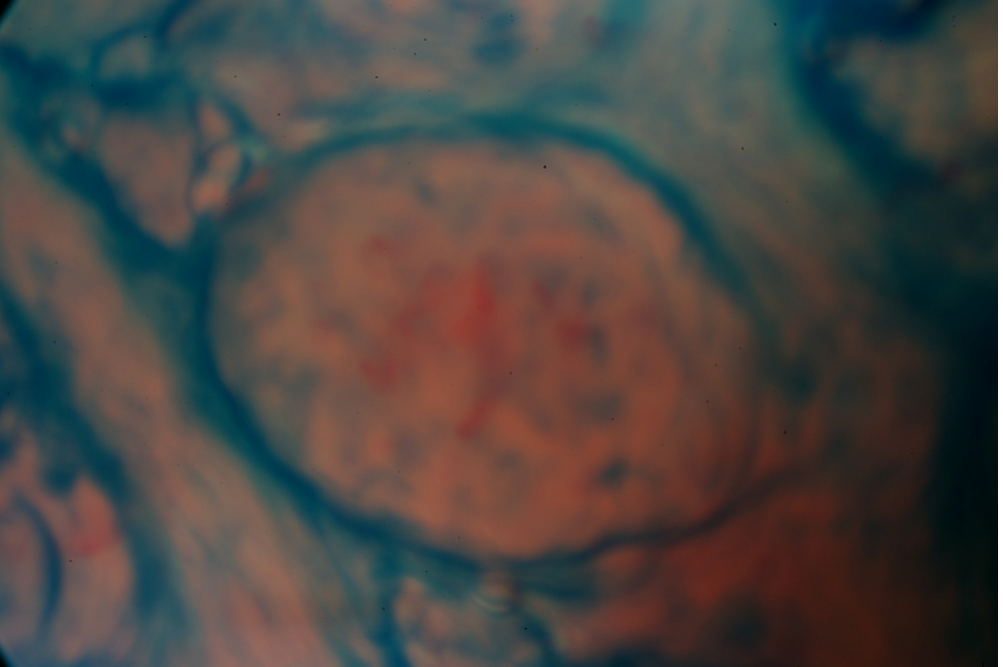
**Taste papillae of a smoker**. They belong to Type 3 (thick and irregular surface) and Type B (elongated blood vessels) corresponding. This smoker used to smoke 20 cigarettes per day the last four years. He never complained for any specific taste disturbance.

In smokers, regions with atrophic papillary structures could be observed. The papillae of 22 smokers' (79%) belonged to **Type 3 **and **Type B**, as seen on Figure 2, and **C **corresponding. Seventeen of them (60%) belonged to **Type B **and five (19%) to **Type C**. The fPap of the six smokers, whose EGM thresholds were low, belonged to **Type 2 **and **B**.

The number of fPap per cm^2 ^at the tongue tip in the two groups is presented in Table 8.

**Table 8:** Number of Fungiform Papillae per cm^2 ^(means ± SD) at Tongue Tip in Smokers and Non-Smokers

	**Non-Smokers**		**Smokers**	
**Tongue tip**	Right Side	Left Side	Right Side	Left Side
	27 ± 7.6	26 ± 6.	25.7 ± 4.2	24.9 ± 5.9

The number of Fungiform Papillae per cm^2 ^(means ± SD) at Tongue Tip in smokers (n = 28) and non-smokers (n = 34). There was no significant difference between the two groups concerning the density of fPap.

## Discussion

The present study focused on people at early age in order to produce unequivocal results. It has been suggested that thresholds measured by EGM increase with age, starting at the age of sixty years, in the areas that are sub served by the chorda tympani and glossopharyngeal nerves and from above the age of seventy years, in the area sub served by the greater petrosal nerve [22].

Several studies have focused on the impact of smoking on taste thresholds. The majority of them were based on the whole-mouth technique and the use of chemical solutions. There is controversy regarding their results. It has been claimed by some authors that no significant impact of smoking on taste sensitivity was found [23,24], while by some others there are contradicting conclusions. In the last category belong authors who reported a higher threshold for detecting bitter taste among the smokers or they found that smoking causes an increase of the recognition threshold for all four basic tastes [25]. Previous studies showed that nicotine is represented as bitter stimulus in the nucleus of solitary tract of the rats [26].

Statistically significant differences were detected among the taste thresholds, as recorded in every one of the four different durations of stimuli in smokers and non-smokers. The present study extends to the findings of previous studies and demonstrates that higher EGM thresholds are also found in younger smokers. The outcoming results agree with these of other researchers who found that EGM thresholds were significantly higher in smokers [27].

Taken for granded that the thresholds were higher for smokers, one might anticipate a different effect of duration for smokers than for non-smokers. Our results show that the greater duration does not result to a higher threshold. As it has been stated in the Results-section there was an increase of the thresholds in all 6 loci when a 1-sec duration stimulus was applied. The thresholds remained almost the same when a 1.5-sec duration stimulus was applied and increased again when the authors used a2-sec duration stimulus. There is controversy concerning the effects of stimulation duration on electrogustometric thresholds. A previous study,(only one subject examined) concludes that electrical thresholds reach asymptote at approximately 1 sec [28]. It has been reported that a group of 9 examined subjects presented a slightly higher median electrical taste threshold for a 750-ms duration stimulus than for a 500 ms stimulus [29]. Some other authors who used the same type of electrogustometer as ours to evaluate the effects of stimulus duration on electrogustometric thresholds of 24 subjects (12 male and 12 female) concluded that the 1-sec duration stimulus resulted to a lower threshold value than the 0.5- and 1.5 sec stimulus which did not differ in magnitude from each other [30]. We believe that it is a subject of further investigation. The difference between the left and the right sides is maintained to 4 dB. A difference of 6 dB or more is indicative of a pathological condition [31].

All of the subjects recognized the stimuli applied as a sour/metallic taste. It should be taken under consideration the evidence that stimuli which deliver >100 uA might be sufficiently strong to activate trigeminal afferents [32]. It is possible that the taste thresholds for some smokers were undefined (probably higher than recorded values) and that these smokers were responding to trigeminal rather than gustatory stimulation.

One finding needing further investigation is that 6 of the smokers (21%) had normal taste thresholds despite the fact that for at least the last two years they smoked a considerable number of cigarettes (~20 per day). It is note-worthy that it had been reported that male smokers in their 30 s and 40 s present with significantly lower thresholds for their soft palate compared to non-smokers [33]. It is possible that both findings are due to changes in the shape and vascularisation which may not be of the same degree in all smokers or that the cell turnover in some of the smokers is similar to that of the non-smokers. This latter hypothesis has been proposed in a previous experimental study, where the long-term effects of nicotine were studied on rat fungiform taste buds [34]. It is also important to remember that the intake of nicotine depends on factors such as the depth and speed of inhalation, the way the cigarette is held, smoking behavior and dependence and nicotine metabolism [34-36].

Electrogustometric threshold assessment has good test-retest reliability but this reliability can be enhanced by the combined use of other methods such as Contact Endoscopy. Indeed, changes in the morphology of papillary structures among smokers and non-smokers were observed with CE.

Despite the significant differences in the shape and the vascularisation of the fPap, there were no differences found concerning the numbers of the papillae of the two groups. It has been suggested that long-term exposure of taste buds to nicotine leads to significant reduction in their size [34]. However any change in shape and size of the papillae is not accompanied by any simultaneous significant change in their number [34]. It has been suggested that taste sensitivity is based on the stimulation area and concentration of the tastant. Our study shows that the density of taste papillae is not the major factor which affects taste acuity. Besides the number and the shape and the vascularisation of the papillae are also important.

This study supports the combination of EGM and CE for the study of taste disorders. Both techniques provide useful clinical data in a short time and have good test-retest reliability. Further study concerning the quantitative measuring system and the determination of histological and morphological parameters is required.

## Conclusion

Statistically important differences between the thresholds of smokers and those of non-smokers were detected. Differences concerning the shape and the vascularisation of fPap were also observed. It is suggested that nicotine causes functional and morphological alterations of papillae, at least in young adults, without severely affecting their number. A finding that needs further investigation is that 21% of the smokers in our sample showed taste thresholds, shape and fPap vessels similar to those of non-smokers. The combined use of EGM and CE can provide useful data about the effects of nicotine on taste function.

## Competing interests

The authors declare that they have no competing interests.

## Authors' contributions

PP carried out the measurements with EGM and CE, drafted the manuscript, and performed the statistical analysis, NV participated in the design of the study, helped to draft the manuscript and to perform the statistical analysis, AA participated in designing the study and drafting the manuscript, KD participated in the design of to draft the manuscript, KG conceived of the study and participated in its design, AG participated in the study's design and coordination and helped to draft the manuscript. All authors read and approved the final manuscript.

## Pre-publication history

The pre-publication history for this paper can be accessed here:


